# Live birth rate and neonatal outcomes of different quantities and qualities of frozen transferred blastocyst in patients requiring whole embryo freezing stratified by age

**DOI:** 10.1186/s12884-020-03353-5

**Published:** 2020-10-29

**Authors:** Shiping Chen, Hongzi Du, Jianqiao Liu, Haiying Liu, Lei Li, Yuxia He

**Affiliations:** 1 Department of Reproductive Medicine, The Third Affiliated Hospital of Guangzhou Medical University, 63 Duobao Road, Liwan District, Guangzhou, China; 2 Key Laboratory of Reproductive Medicine of Guangdong Province, Guangdong Guangzhou, China; 3 Key Laboratory for Major Obstetric Diseases of Guangdong Province, Guangzhou, Guangdong China; 4 Key Laboratory of Reproduction and Genetics of Guangdong Higher Education Institutes, Guangzhou, Guangdong China

**Keywords:** Blastocyst quality, Blastocyst quantity, Double blastocyst transfer, Single blastocyst transfer, Live birth rate, Neonatal outcomes

## Abstract

**Background:**

Multiple pregnancies are associated with significant complications and health risks for both mothers and infants. Single blastocyst transfer (SBT) is a logical and effective measure to reduce the incidence of multiple pregnancy with assisted reproductive technology (ART). Whether it is suitable for everyone undergoing SBT was inconclusive, in view of the consideration of embryo quality and patients’ age. Therefore, this study aimed to explore live birth rate (LBR) and neonatal outcomes of different quantities and qualities of blastocysts in patients stratified by age, using a cutoff of 35 years, who required whole embryo freezing and underwent a subsequent frozen thawed transfer (FET) cycle.

**Methods:**

Atotal of 3,362 patients were divided into five groups: group A (*n*=1569) received a single good-quality blastocyst, group B (*n*=1113) received two good-quality blastocysts, group C (*n*=313) received one good-and one average-quality blastocyst, group D (*n*=222) received two average-quality blastocysts, and group E (*n*=145) received one average-quality blastocyst.

**Results:**

For patients who received good-quality blastocysts, irrespective of age, the LBR of double blastocyst transfer (DBT) was about 50–65% and the multiple pregnancy rate (MPR) was 40–60%; however, the LBR of SBT was 40–55%, and the MPR was 3.5–6.3%. For patients who only had average-quality blastocysts, the MPR of double average-quality blastocyst transfer was as high as 30–50%. Moreover, about 70–90% of preterm births resulted from multiple pregnancies, and about 85–95% of low birth weight babies come from multiple pregnancies. The neonatal outcomes (gestational age, birth weight, and birth height) of DBT were significantly lower than those of SBT regardless of age, and this statistical difference disappeared if the patients were subgrouped by singleton or twin. There is no significant difference in neonatal outcomes between single good-quality blastocyst and single average-quality blastocyst transfer.

**Conclusions:**

SBT is a preferable option for patients regardless of age when good-quality blastocysts are available. For patients who only had average-quality blastocysts, they should be informed that DBT was associated with higher multiple pregnancy and adverse neonatal outcomes when compared with SBT regardless of age, suggesting that the practice of SBT is also feasible for these patients.

##  Background

Since the birth of the world’s first baby with the help of in vitro fertilization-embryo transfer (IVF-ET) in 1978 [1], this technology has become an effective procedure for infertile patients and is widely used worldwide. Two or more embryos were transferred to increase the chance of pregnancy, as a result, leading to a high risk of multiple pregnancy, which is considered as the most common adverse event associated with IVF-ET technology [2]. Multiple pregnancies are associated with significant complications and health risks for both mothers and infants [3]. Therefore, clinicians are gradually shifting from the initial goal of obtaining pregnancy to attaining the birth of a single healthy baby. Obviously, decreasing the number of transferred embryos, specifically, employing the practice of single embryo transfer, is a logical and effective measure to reduce the incidence of multiple pregnancy with assisted reproductive technology (ART) [4, 5].

With the improvement of laboratory environments and culture conditions, embryo culture can be extended to the blastocyst stage. Blastocyst culture has the advantage of self-selection of viable embryos that attain a greater implantation rate and pregnancy outcomes. A previous study showed a significantly higher pregnancy rate in patients undergoing single blastocyst transfer (SBT) versus single cleavage stage embryo [6]. Another study indicated that selective SBT (eSBT) significantly reduced the risk of multiple pregnancy without compromising the pregnancy rate compared with double blastocyst transfer (DBT) [7, 8].

A clear definition of selective SBT was given in a study [9], namely, a single blastocyst was transferred, and at least one blastocyst was available for cryopreservation. However, the detailed grading of transferred embryos for eSBT and DBT in these studies was not clearly described [7, 10, 11]. The quality of the blastocysts transferred in the DBT group met the conditions of one of the three scenarios: the two blastocysts may have been both of good-quality, average-quality, or a combination of one good- and one average-quality blastocyst. As far as we know, there have been no relevant studies stratified by age that compare the live birth rate (LBR) and neonatal outcomes derived from the three cases of DBT with single good-quality blastocyst transfer. The purpose of this study was to evaluate the pregnancy and neonatal outcomes of different numbers and grades of frozen blastocysts in patients stratified by age who required whole embryo freezing and underwent a subsequent frozen-thawed transfer cycle. Ultimately, this information will provide strong evidence for clinicians who select the number of transferred embryos based on maternal age and embryo quality in clinical practice.

##  Methods

### Study population and grouping

This was a retrospective, single-center study of patients undergoing frozen embryo transfer (FET) from January 2016 to October 2018 at the Department of Reproductive Medicine Center in the Third Affiliated Hospital of Guangzhou Medical University, Guangzhou, China. Inclusion criteria included the following: (1) women 20 to 42 years of age, (2) basal follicle-stimulating hormone (FSH) < 10 mIU/mL, (3) first IVF/ICSI (intracytoplasmic sperm injection) cycle, (4) first FET cycle after whole embryo freezing, (5) transfer of one or two day 5 blastocysts, and (6) endometrium ≥ 7 mm. The reasons for whole embryo freezing included the prevention of moderate and severe OHSS (ovarian hyperstimulation syndrome), increased progesterone level on human chorionic gonadotrophin (HCG) day, untreated hydrosalpinx, and personal reasons (acute internal, surgical and gynecological diseases, and non-medical factors). Exclusion criteria included the following: (1) donated oocytes or embryos; (2) cycles with preimplantation genetic testing (PGT); (3) known uterine anomalies included intrauterine adhesion, septal uterine cavity, endometrial polyps, submucosal fibroid, etc.; (4) untreated hydrosalpinx surgically prior to FET; (5) stage III–IV endometriosis or adenomyosis, and (6) uncontrolled endocrine and/or immune disorders or other systemic diseases, including hypertension, diabetes, thyroid disease, hyperprolactinemia, antiphospholipid syndrome, systemic lupus erythematosus, etc. Each patient signed an informed consent upon obtaining and analyzing their clinical data prior to the initiation of IVF/ICSI-ET treatment.

These women, aged 20–42 years old, were divided into five groups depending on the quantity and quality of the day 5 blastocyst: group A (*n* = 1569) received one good-quality blastocyst, group B (n = 1113) received two good-quality blastocysts, group C (*n* = 313) received one good- and one average-quality blastocysts, group D (*n* = 222) received two average-quality blastocysts, and group E (*n* = 145) received one average-quality blastocyst.

### Ovarian stimulation

Patients underwent controlled ovarian stimulation with either a long protocol using gonadotrophin-releasing hormone (GnRH) agonist (triptorelin, Diphereline®, Ipsen, France) or GnRH antagonist (cetrorelix, Cetrotide®, Merck, Germany) protocol as previously described [12]. Individually determined dosages of recombinant human follitropin (r-hFSH; GONAL-*f* ®, Merck Serono, Switzerland, or Puregon®, MSD, Netherlands) were performed, and follicular development were monitored using transvaginal ultrasonography and serum oestradiol determinations. At least three follicles ≥ 17 mm was required to induce oocyte maturation using urine human chorionic gonadotrophin (uHCG, Lizhu Group Co., China) or recombinant HCG (rHCG, Merck Serono). Oocyte retrieval was performed 36–38 h after administration of HCG, and oocytes were incubated for insemination by conventional IVF or ICSI as determined by sperm quality.

### Embryo grading, vitrification, and warming

Blastocysts were graded and scored using the Garden criteria [13] according to blastocyst expansion, inner cell mass (ICM) development, and trophectoderm (TE) appearance. Patients in our study were only transferred day 5 blastocyst. Blastocysts graded 4 and over with ICM A or B and TE A or B were considered as good-quality embryo. Those blastocysts that presented as grade 4 and over with an ICM C or TE C and all blastocysts that graded 3 were regarded as average-quality embryos.

All available blastocysts were cryopreserved by vitrification method according to manufacturer’s instruction. Blastocyst warming was performed by a rapid thawing method in the morning of embryo transfer. The number and stage of transferred embryos were determined by clinicians and patients, giving priority to patients’ age, blastocyst qualities and quantities.

### Frozen-thawed cycle and embryo transfer

Endometrial preparations for FET including the natural cycle (NC) program and hormone replacement therapy (HRT) program have been described previously [12]. In short, the NC program was suitable for patients with a regular menstrual cycle and ovulation. One or two blastocysts were transferred on the sixth day after ovulation. HRT was applicable in patients with irregular menstrual cycles or poor endometrium development in NC. The endometrium preparation of HRT used daily oral estradiol valerate tablets (Progynova®, Bayer, Germany) from the second to the fourth day after menstruation, and embryo transfer was performed on the sixth day of progesterone (60 mg/day) injection. All patients received luteal support with progesterone after embryo transfer until 10 weeks after conception.

### Statistical analysis

We used the Statistical Package for Social Science (SPSS) version 22.0 software for statistical analysis. The continuous variables were expressed as the mean ± standard deviation (SD) and compared by one-way analysis of variance (ANOVA) or Student’s t-test. Categorical data were described as frequencies and percentages and compared using chi-square test or Fisher’s exact test. A two-sided *P* value of 0.05 or less was considered statistically significant. A baby born alive after 28 weeks gestation was classified as a live birth. Clinical pregnancy was confirmed by ultrasonographic visualizaiton of gestational sac 4–5 weeks after embryo transfer, and low birth weight was defined as birth weight less than 2,500 g and very low birth weight less than 1,500 g.

##  Results

A total of 3,362 patients who met the inclusion criteria were included in the study from January 2016 to October 2018. A total of 22 pregnant patients were lost to follow-up in this study. The clinical and neonatal outcomes were analyzed according to age, using a cutoff of 35 years. The patients’ baseline characteristics are presented in Table 1. When patients were in the same age category, no significant differences were found in terms of age, body mass index (BMI), basal FSH, anti-mullerian hormone (AMH), infertility duration, type of infertility, type of endometrial preparation, and endometrium thickness (all values of *P *> 0.05).

**Table 1:** Baseline characteristics of patients among groups A–E stratified by age

	<35 years of age						≥ 35 years of age					
	Group A	Group B	Group C	Group D	Group E	*P*	Group A	Group B	Group C	Group D	Group E	*P*
Cycles (n)	1425	844	206	183	120		144	269	107	39	25	
female age(year)	29.17 ± 2.89	29.32 ± 2.92	29.45 ± 2.93	29.50 ± 2.69	29.38 ± 3.62	0.419	36.65 ± 1.91	37.17 ± 2.01	37.16 ± 1.93	37.51 ± 2.53	37.08 ± 2.57	0.063
BMI (kg/m2)	21.46 ± 3.09	21.70 ± 3.20	21.34 ± 3.13	21.71 ± 3.48	21.67 ± 3.41	0.343	21.96 ± 2.75	22.59 ± 2.82	22.59 ± 2.88	22.38 ± 2.45	22.06 ± 2.41	0.210
Basal FSH (mIU/ml)	5.08 ± 1.24	4.92 ± 1.25	5.07 ± 1.37	4.96 ± 1.21	5.16 ± 1.44	0.104	5.15 ± 1.63	5.15 ± 1.31	4.81 ± 1.22	5.15 ± 1.66	5.11 ± 0.91	0.701
AMH (ng/ml)	8.06 ± 5.71	7.80 ± 4.03	7.22 ± 3.68	6.85 ± 3.72	6.98 ± 3.80	0.095	6.08 ± 3.56	6.34 ± 3.75	5.08 ± 2.25	5.74 ± 3.86	5.80 ± 3.07	0.347
Duration of infertility (years)	3.44 ± 1.94	3.57 ± 2.06	3.85 ± 2.08	3.53 ± 1.94	3.33 ± 1.89	0.052	4.21 ± 3.39	5.08 ± 3.92	5.06 ± 3.96	5.81 ± 4.17	4.72 ± 3.02	0.099
Type of infertility						0.276						0.522
Primary infertility	56.14 (800/1425)	52.25 (441/844)	56.31 (116/206)	55.19 (101/183)	60.83 (73/120)		27.08 (39/144)	33.09 (89/269)	25.23 (27/107)	33.33 (13/39)	28.0 (7/25)	
Secondary infertility	43.86 (625/1425)	47.75 (403/844)	43.69 (90/206)	44.81 (82/183)	39.17 (47/120)		72.92 (105/144)	66.91 (180/269)	74.77 (80/107)	66.67 (26/39)	72.0 (18/25)	
Endometrial preparation (%)						0.669						0.149
HRT	84.0 (1197/1425)	86.26 (728/844)	85.44 (176/206)	84.70 (155/183)	83.33 (100/120)		70.83 (102/144)	76.95 (207/269)	82.24 (88/107)	79.49 (31/39)	64.0 (16/25)	
Natural	16.0 (228/1425)	13.74 (116/844)	14.56 (30/206)	15.30 (28/183)	16.67 (20/120)		29.17 (42/144)	23.05 (62/269)	17.76 (19/107)	20.51 (8/39)	36.0 (9/25)	
Endometrial thickness (mm)	9.04 ± 1.61	8.67 ± 1.58	8.85 ± 1.53	8.93 ± 1.60	8.99 ± 1.56	0.106	8.98 ± 1.83	8.89 ± 1.61	8.96 ± 1.63	8.86 ± 1.73	9.13 ± 1.52	0.939

*BMI* body mass index, *FSH* follicle-stimulating hormone, *AMH* anti-mullerian hormone, *HRT* hormone replacement therapy

group A, one good-quality blastocyst; group B, two good-quality blastocysts; group C, one good- and one average-quality blastocyst; group D, two average-quality blastocysts, group E, one average-quality blastocyst

The clinical outcomes of the patients in groups A–E stratified by 35 years of age are shown in Table 2. There were significant differences in the rates of implantation, clinical pregnancy, live birth, and multiple pregnancy among groups A–D when patients were in the same age category. For women under 35 years old, the LBR in groups B and C was significantly higher than group A, but the LBR in group A was acceptable (54.2%), and the multiple pregnancy rate (MPR, 3.5%) was significantly lower than groups B (62.4%) and C (49.7%). There was no significant difference in the LBR between groups D and A, but the MPR in group D was significantly higher than that in group A (50% vs. 3.5%). In women 35 years of age and older, the LBR (which still reached 42.4%) and MPR (6.3% vs. 49.2%) in group A were significantly lower than that in group B. There was no significant difference in the LBR between groups A and C or D, but the MPR in group A was significantly lower than that in groups C and D. Meanwhile, we compared the clinical outcomes of patients with two average-quality blastocysts (group D) versus one average-quality blastocyst (group E). The results showed that for patients under 35 years old, the LBR in group D was higher than that in group E, but the MPR was as high as 50% (*P *< 0.01). In women 35 years of age and older, there was no significant difference in the LBR and MPR between groups D and E; however, patients in group D had a nonsignificant trend toward a higher multiple rate than group E (31.3% vs. 10%).

**Table 2:** The clinical outcomes of patients in groups A–E stratified by 35 years of age

	<35 years of age						≥ 35 years of age					
	Group A	Group B	Group C	Group D	Group E	*P*^*#*^	Group A	Group B	Group C	Group D	Group E	*P*^*#*^
Cycles (n)	1425	844	206	183	120		144	269	107	39	25	
Implantation rate	63.72^b,c^(908/1425)	60.37(1019/1688)	53.64(221/412)	45.90(168/366)	46.67(56/120)	0.000	55.56^b,c^(80/144)	52.97(285/538)	40.19(86/214)	26.92(21/78)	40.0(10/25)	0.000
Pregnancy rate	63.72^a,b^(908/1425)	75.59(638/844)	72.33(149/206)	62.30(114/183)	46.67^d^(56/120)	0.000	55.56^a^(80/144)	73.23(197/269)	57.0(61/107)	41.03(16/39)	40.0(10/25)	0.000
Live birth	54.25^a,b^(773/1425)	64.57 (545/844)	64.08 (132/206)	48.63 (89/183)	36.67 ^d^(44/120)	0.000	42.36^a^(61/144)	59.48(160/269)	48.60(52/107)	30.77(12/39)	24.0(6/25)	0.000
Singleton	52.70^a,b,c^ (751/1425)	33.05(279/844)	38.84(80/206)	25.13(46/183)	36.67^d^ (44/120)	0.000	40.97 (59/144)	38.29(103/269)	33.64(36/107)	23.08(9/39)	24.0 (6/25)	0.177
Twin	1.55^a,b,c^ (22/1425)	31.52(266/844)	25.24(52/206)	23.50 (43/183)	0.0^d^ (0/120)	0.000	1.39^a,b^ (2/144)	21.19 (57/269)	14.95 (16/107)	7.69 (3/39)	0.0 (0/25)	0.000
Multiple pregnancy rate	3.52^a,b,c^ (32/908)	62.38 (398/638)	49.66 (74/149)	50.0 (57/114)	0.0^d^ (0/56)	0.000	6.25^a,b,c^ (5/80)	49.24 (97/197)	42.62 (26/61)	31.25 (5/16)	10.0 (1/10)	0.000
Monozygotic twins rate	3.52 (32/908)	2.66 (17/638)	1.34 (2/149)	2.63 (3/114)	0.0 (0/56)	0.462	6.25 (5/80)	4.57 (9/197)	1.64 (1/61)	0.0 (0/16)	10.0 (1/10)	0.462
Early abortion rate	11.67^a^ (106/908)	8.31 (53/638)	8.72 (13/149)	15.79 (18/114)	21.43 (12/56)	0.037	20.0 (16/80)	17.26 (34/197)	13.11 (8/61)	18.75 (3/16)	30.0 (3/10)	0.758
Abortion rate	13.33 (121/908)	11.44 (73/638)	9.40 (14/149)	19.30 (22/114)	21.43 (12/56)	0.067	21.25 (17/80)	18.78 (37/197)	14.75 (9/61)	25.0 (4/16)	30.0 (3/10)	0.715
Ectopic pregnancy rate	0.88 (8/908)	1.25 (8/638)	1.34 (2/149)	0.0 (0/114)	0.0 (0/56)	0.597	2.50 (2/80)	0.0 (0/197)	0.0 (0/61)	0.0 (0/16)	10.0 (1/10)	0.076

^*#*^comparison among groups A–D. *P* < .05 was considered statistically significant

^a^*P* < .05 compared to group B

^b^*P* < .05 compared to group C

^c^*P* < .05 compared to group D

^d^*P* < .05 between groups D and E

Comparisons of neonatal outcomes of patients among groups A–E stratified by 35 years of age are presented in Table 3. The results showed that 70–90% of preterm births resulted from multiple pregnancies, and about 85–95% of low birth weight babies come from multiple pregnancies. When patients were in the same age category, the gestational age, birth weight, and birth height of group A were significantly higher than those in groups B, C, or D, but this statistical difference disappeared if the patients were subgrouped by singleton or twin birth. Meanwhile, there was no significant difference in terms of the gestational age, birth weight, and birth height between groups A and E. Finally, the neonatal outcomes were compared between groups D and E, and the results showed that the gestational age, birth weight, and birth height of patients in group D were significantly lower than those in group E when patients were in the same age category.

**Table 3:** The neonatal outcomes of patients among groups A–E stratified by 35 years of age

	<35 years of age						≥ 35 years of age					
	Group A	Group B	Group C	Group D	Group E	*P*	Group A	Group B	Group C	Group D	Group E	*P*
Cycles (n)	1425	844	206	183	120		144	269	107	39	25	
Preterm birth (<37weeks)	11.77^a,b,c^ (91/773)	39.45 (215/545)	33.33 (44/132)	42.70 (38/89)	4.55^d^ (2/44)	0.000	8.20^a,b^ (5/61)	35.0 (56/160)	25.0 (13/52)	16.67 (2/12)	16.67 (1/6)	0.001
Singleton	8.67^a^ (67/773)	3.30 (18/545)	6.82 (9/132)	3.37 (3/89)	4.55 (2/44)	0.001	4.92 (3/61)	5.62 (9/160)	7.69 (4/52)	0.0 (0/12)	0.0 (0/6)	0.755
Twin	3.10^a,b,c^ (24/773)	36.15 (197/545)	26.51 (35/132)	39.33 (35/89)	0.0^d^ (0/44)	0.000	3.28^a,b^ (2/61)	29.38 (47/160)	17.31 (9/52)	16.67 (2/12)	16.67 (1/6)	0.000
Gestational age (weeks)	38.70 ± 1.94^a,b,c^	37.37 ± 2.52	37.59 ± 2.52	36.86 ± 3.33	39.18 ± 1.91^d^	0.000	38.72 ± 1.23^a,b,c^	37.61 ± 2.33	37.66 ± 2.89	37.00 ± 3.43	38.32 ± 0.86^d^	0.022
Singleton	38.80 ± 1.83	38.94 ± 1.71	38.51 ± 2.18	39.00 ± 1.58	39.18 ± 1.91	0.325	38.81 ± 1.56	38.59 ± 2.14	38.76 ± 1.97	38.52 ± 1.71	38.32 ± 0.86	0.951
Twin	35.32 ± 1.47	36.04 ± 2.32	36.02 ± 2.37	35.15 ± 3.01	/	0.095^*#*^	36.20 ± 0.40	35.98 ± 1.63	35.06 ± 3.21	34.29 ± 4.99	/	0.330^*#*^
Birth height (mm)	49.51 ± 2.3^a,b,c^	47.72 ± 3.21	48.23 ± 2.41	46.61 ± 4.26	49.89 ± 2.22^d^	0.000	49.77 ± 1.85^a,b,c^	48.03 ± 2.70	47.95 ± 3.75	48.31 ± 5.70	49.75 ± 0.96^d^	0.000
Singleton	49.68 ± 2.27	49.82 ± 1.98	49.88 ± 1.73	49.53 ± 1.44	49.88 ± 2.22	0.838	49.93 ± 1.76	49.50 ± 2.39	49.77 ± 2.41	50.00 ± 2.00	49.75 ± 0.96	0.868
Twin	46.66 ± 2.40	46.72 ± 2.95	46.97 ± 2.20	45.50 ± 4.32	/	0.113^*#*^	46.75 ± 0.35	46.90 ± 1.91	45.70 ± 3.70	45.83 ± 6.83	/	0.155^*#*^
Birth weight (kg)	3.16 ± .054^a,b,c^	2.73 ± 0.61	2.79 ± 0.62	2.57 ± 0.66	3.19 ± 0.43^d^	0.000	3.12 ± 0.51^a,b,c^	2.76 ± 0.60	2.81 ± 0.75	2.55 ± 0.88	3.30 ± 0.29^d^	0.000
Singleton	3.22 ± 0.50	3.27 ± 0.51	3.24 ± 0.52	3.17 ± 0.33	3.19 ± 0.43	0.583	3.36 ± 0.49	3.22 ± 0.50	3.24 ± 0.49	3.26 ± 0.45	3.30 ± 0.29	0.666
Twin	2.34 ± 0.43	2.48 ± 0.45	2.44 ± 0.42	2.31 ± 0.54	/	0.113^*#*^	2.55 ± 0.25	2.43 ± 0.34	2.24 ± 0.52	2.00 ± 0.82	/	0.142^*#*^
Still birth	0.0 (0/773)	0.0 (0/545)	0.0 (0/132)	0.0 (0/89)	0.0 (0/44)	/	0.0 (0/61)	0.0 (0/160)	0.0 (0/52)	0.0 (0/12)	0.0 (0/6)	/
Congenital anomalies	1.64 (13/795)	1.60 (13/811)	1.09 (2/184)	1.52 (2/132)	2.27 (1/44)	0.958	0.0 (0/63)	0.0 (0/217)	0.0 (0/68)	0.0 (0/15)	0.0 (0/6)	/
Sex ratio(male/female)	1.30 (450/345)	1.43 (477/334)	1.10 (97/87)	1.46 (78/54)	0.83 (20/24)	0.445	1.43 (37/26)	1.22 (119/98)	1.16 (37/31)	0.63 (6/9)	1.00 (3/3)	0.632
Low birth weight (<2500 g) in singleton	7.04^a,b,c^ (56/795)	27.25 (221/811)	28.26 (52/184)	34.09 (45/132)	4.55^d^ (2/44)	0.000	6.34^a,b^ (4/63)	29.03 (63/217)	27.94 (19/68)	13.33 (2/15)	0.0 (0/6)	0.002
Singleton	4.02 (32/795)	4.44 (36/811)	4.35 (8/184)	4.55 (6/132)	4.55 (2/44)	0.977	3.17 (2/63)	2.76 (6/217)	1.47 (1/68)	0.0 (0/1)	0.0 (0/6)	0.833
Twin	3.02^a,b,c^ (24/795)	22.81 (185/811)	23.91 (44/184)	29.54 (39/132)	0.0^d^ (0/44)	0.000	3.17^a,b^ (2/63)	26.27 (57/217)	26.47 (18/68)	13.33 (2/15)	0.0 (0/6)	0.001
Very low birth weight (<1500 g) in singleton	0.75^a,c^ (6/795)	2.84 (23/811)	1.63 (3/184)	5.30 (7/132)	0.0 (0/44)	0.001	0.0^c^ (0/63)	2.76 (6/217)	2.94 (2/68)	13.33 (2/15)	0.0 (0/6)	0.045
Singleton	0.63 (5/795)	0.12 (1/811)	0.54 (1/184)	0.0 (0/132)	0.0 (0/44)	0.324	0.0 (0/63)	0.5 (1/217)	0.0 (0/68)	0.0 (0/15)	0.0 (0/6)	0.879
Twin	0.12^a,c^ (1/795)	2.72 (22/811)	1.09 (2/184)	5.30 (7/13)	0.0 (0/44)	0.000	0.0^c^ (0/63)	2.30 (5/217)	2.94 (2/68)	13.33 (2/15)	0.0 (0/6)	0.029

^*#*^comparison among groups A–D. *P* < .05 was considered statistically significant

^a^*P* < .05 compared to group B

^b^*P* < .05 compared to group C

^c^*P* < .05 compared to group D

^d^*P* < .05 between groups D and E

## Discussion

The suggestion on the number of transferred embryos is usually affected by the patient’s age in clinical practice. Doctors tend to recommend transferring two embryos for older patients to increase the clinical pregnancy rate. It has been reported that the LBR is similar in the transfer of one or two blastocysts [14]. However, it is not certain whether the above conclusions are applicable to older patients. There are two studies that explore the effect of the number of blastocyst transfer on pregnancy outcomes subgrouped by age. Eum and colleagues found that the live birth or ongoing pregnancy rate of eSBT and DBT was equivalent, but eSBT had a lower risk of multiple pregnancy, regardless of age, for both fresh and vitrified-warmed cycles [7]. Similarly, another investigation reported a comparable pregnancy rate and a significantly reduced MPR of eSBT compared to that of DBT in patients 35–39 years of age [15]. Nevertheless, it is worth emphasizing that transferred blastocyst quality has not been mentioned in detail in these studies, which cannot be ignored for the embryo quality is also a determinant factor of success in ART cycles. Therefore, this study is the first to explore the pregnancy and neonatal outcomes associated with different quantities and qualities of blastocysts transferred in patients undergoing FET cycles after whole embryo freezing stratified by age. Our results showed that SBT is a preferable strategy for patients irrespective of age when good-quality blastocysts are available. For patients who only had average-quality blastocysts, be cautious about suggesting the transfer of two embryos because DBT was associated with higher multiple pregnancy and adverse neonatal outcomes when compared with SBT even for older patients.

A woman’s age is considered the most important factor that influences fertility potential, and this potential significantly decreases after the age of 35 years [16]. Extending embryo culture to the blastocyst stage has the advantage of natural selection of the most viable, genetically competent embryos, which is particularly important for advanced maternal age [17]. A recent prospective study reported a higher ongoing pregnancy rate following blastocyst transfer than cleavage embryo transfer in women 35 years of age and older, whereas the difference was not significant in younger women [18]. An increased risk of adverse obstetrical and neonatal complications associated with multiple pregnancy was observed, especially for patients of advanced maternal age [19]. Our results suggested that SBT also appeared to be a promising option that did not compromise the LBR for women over the age of 35 years with available good-quality blastocysts. Moreover, recent studies also indicated that the practice of selective SBT was feasible and resulted in reduced MPRs in women aged 40–43 years without compromising cumulative LBR compared with DBT [9, 10], suggesting that maternal age was not a significant predictor of live birth, and the competence of the oocytes developing into good-quality blastocysts is more important than maternal age (10). Additionally, another study reported that maternal age has no effect on pregnancy rates when fully expanded blastocysts are achieved [20]. Therefore, we believe that attaining more, good-quality blastocysts through the improvement of the stimulation protocol and culture environment is crucial for older women.

To increase the odds of a successful pregnancy in patients without good-quality blastocysts, two blastocysts were usually transferred to patients in our reproductive center. However, this strategy keeps the MPR per year at around 30% in patients undergoing FET in our reproductive center before 2018. Our study indicated that in these women with DBT, the MPR was as high as 50% in patients under 35 years of age and 31.3% in patients aged 35 years and over. A previous study has highlighted that the multiple birth rate is 28% in women aged 38–40 years when two embryos are transferred [21], suggesting that advanced maternal age does not protect against multiple pregnancy. In advanced women who only has average-quality blastocyst, our results showed that the clinical PR and LBR observed in patients with SBT were similar to that of DBT, and no multiple pregnancies occurred with SBT. Because these results were obtained based on the small sample size of patients, it is difficult to advocate a routine policy of SBT in patients over the age of 35 years without good-quality blastocysts. However, couples with only average-quality blastocysts should be informed that DBT can obtain an MPR of 30–50%, which leads to higher perinatal morbidity and mortality rates than those associated with single embryo transfer [4, 9]. Additionally, our results were in accordance with the guidelines of the American Society for Reproductive Medicine, which recommends that eSBT should be performed in patients under 35 years old with good prognosis and should also be considered in women aged 35–42 if they have good-quality euploid blastocysts available for transfer [22]. In view of the results of this study, regardless of age, we recommend and encourage patients with D5 blastocysts to conduct SBT. The MPR is about 15% on 2019 in our center, and the clinical PR has not been significantly affected. Therefore, we believe that this study’s conclusion is very important and has clinical significance for doctors and patients.

It is well known that multiple pregnancies are associated with a higher risk of neonatal and perinatal complications [10]. Our results consisted of the aforementioned conclusion, showing that 70–90% of preterm births resulted from multiple pregnancies, and about 85–95% of low birth weight babies come from multiple pregnancies. However, whether embryo quality affects the neonatal outcomes is still controversial. Two previously published studies found that singletons derived from poor-quality embryos were not at a higher risk of adverse neonatal outcomes, and embryo quality was not correlated with pregnancy complications [23, 24]. Our results are in line with these studies describing that there was no difference in the birth weight of newborns between groups A and E. However, a recent study suggested that the transfer of a poor-quality blastocyst was associated with lower mean birth weight when compared with the transfer of an excellent-quality blastocyst during FET cycles [25]. The differences in terms of the study population and degree of embryo development may account for the inconsistent results.

Our study has some limitations that need to be taken into consideration. The retrospective nature of this study is a major limitation; however, it is important to note that there were no differences with regard to patients’ baseline characteristics among groups A–E stratified by age, suggesting that these five cohorts comprised similar populations in this study. The large variation in the number of cases among these groups was another weakness of the study, especially a very small sample of patients with only average-quality blastocyst for the preferential selection of good-quality blastocysts in our study. However, it is worth mentioning that the sample size of patients in this study was larger than those of other similar studies, so the results from the present study are valuable in guiding clinical practice and encouraging SBT in patients undergoing ART when the expanded blastocyst is obtained, especially for patients of advanced maternal age.

## Conclusions

Our results suggested that when good-quality blastocysts are available, SBT should be incorporated into daily practice because of reduced risk of multiple pregnancies without significantly affecting the LBR. In patients who only have average-quality blastocysts, DBT was associated with higher multiple pregnancies and adverse neonatal outcomes when compared with SBT, suggesting that the practice of SBT is also a preferable option in these patients regardless of age.

## Data Availability

The data sets used and/or analyzed during the current study are available from the corresponding author on reasonable request.

## Abbreviations

AMH: Anti-mullerian hormone
ANOVA: One-way analysis of variance
ART: Assisted reproductive technique
BMI: Body mass index
DBT: Double blastocyst transfer
FET: Frozen embryo transfer
FSH: Follicle stimulating hormone
GnRH: Gonadotrophin releasing hormone
HCG: Human chorionic gonadotrophin
HRT: Hormone replacement therapy
ICM: Inner cell mass
ICSI: Intracytoplasmic sperm injection
IVF-ET: In vitro fertilization and embryo transfer
LBR: Live birth rate
MPR: Multiple pregnancy rate
NC: Natural cycle
OHSS: Ovarian hyperstimulation syndrome
PGT: Preimplantation genetic testing
SBT: Single blastocyst transfer
SD: Standard deviation
SPSS: Statistical package for social science
TE: Trophectoderm
